# A modified approach for elbow arthroscopy using an adjustable arm holder

**DOI:** 10.1186/s13018-016-0509-4

**Published:** 2017-01-31

**Authors:** Alvin Chao-Yu Chen, Chun-Jui Weng, Chih-Hao Chiu, Shih-Sheng Chang, Chun-Ying Cheng, Yi-Sheng Chan

**Affiliations:** Bone and Joint Research Center, Department of Orthopaedic Surgery, Chang Gung Memorial Hospital-Linkou and University College of Medicine, 5th, Fu-Shin St., Kweishan Dist Taoyuan, 333 Taiwan, Republic of China

## Abstract

**Background:**

Position shifting from elbow arthroscopy to open surgery could complicate the surgical procedures; patient safety and risks of contamination are of concern. The aim of this study is to retrospectively assess the safety and efficacy of elbow arthroscopy in 32 elbows of 32 patients using a modified arm holder to facilitate subsequent open surgery in supine position.

**Methods:**

We performed a retrospective study in arthroscopy of the elbow performed with patients in the supine position under general or regional anesthesia. Arthroscopic indications were intraarticular lesions with or without second disorders. The operated arm was securely supported using an adjustable arm holder, which allowed a second surgical procedure without repositioning the patient. We recorded arthroscopic findings, clinical outcome, and complications for all patients. The average duration of follow-up was 17.1 months. Functional assessment was based on the Mayo Elbow Performance Score.

**Results:**

All patients had either good or excellent results with a mean Mayo Elbow Performance Score of 89.2 ± 7.2. Final motion arc averaged 113.3 ± 11.8; residual motion limitation was noted in 2 patients with preoperative ankylosis. No complications were observed immediately after surgery or during follow-up except transient paresthesia along medial cutaneous nerve in 2 patients. A total of 17 patients (53.1%) underwent other surgeries (19 procedures) after arthroscopy; 16 of these surgeries were open elbow procedures including ligament repair (7), ligament reconstruction (5), and ulnar nerve transposition (4). The average time for arthroscopy was 45.2 min; the time interval between the end of arthroscopy and the start of the second surgery procedure averaged 6.5 min.

**Conclusions:**

Arthroscopy of the elbow using an adjustable arm holder with the patients in the supine position was safe and efficacious. This procedure eliminates the need for repositioning the patient and thus may facilitate subsequent concomitant surgical procedures.

**Electronic supplementary material:**

The online version of this article (doi:10.1186/s13018-016-0509-4) contains supplementary material, which is available to authorized users.

## Background

The concept and technique of elbow arthroscopy was proposed by Burman in 1932 [1]. The indications for elbow arthroscopy have steadily increased over the past three decades with advancements in the equipment and techniques and the increased clinical experience [2, 3]. Some modifications have been made to the original method to improve its safety and efficacy including portal standardization, nerve identification, distraction device, and patient positioning [4, 5]. With diagnostic arthroscopy of the elbow being used frequently in clinical practice, surgical management of more complex problems was also attempted [6]. Bearing the advantages of similar anatomical orientation to open procedures, supine position recently becomes a popular option for both diagnostic and interventional elbow arthroscopies [7]. However, both classical and currently modified supine position entails several disadvantages including either unstable suspension or elbow flexion fixed in 90°, difficult access to the posterior compartment, and time consumed in conversion to open surgery. We developed a straightforward and effective method using an easily assembled arm holder with the patient in a supine position and examined the effectiveness and safety of the method in 32 patients.

We hypothesized that elbow arthroscopy in a supine position using this adjustable arm holder was a feasible and an efficacious option for both diagnostic purposes and for concomitant reconstruction surgery.

## Materials and methods

We conducted a retrospective study of the arthroscopic surgery in supine position, which was started since early 2007 in our hospital while an adjustable arm holder was not yet applied until 2009. All the patients with the index surgery between July 2009 and June 2011 who were operated by one surgeon (AC Chen) were recruited for this study. Institutional review board approval (104-7280B) was obtained for a review of patients’ records and radiographs. Complete medical records including demographic data, surgical procedures, and functional survey with regular follow-up for at least 1 year were collected in 32 patients (32 elbows). There were 8 women (25%) and 24 men (75%); mean age, 34.4 years (range, 18 to 57 years). Arthroscopic indications based on the primary diagnosis included instability, posterior impingement, loose body, articular fracture, refractory lateral epicondylitis, posttraumatic arthrofibrosis, and osteochondritis dissecans (OCD). A second concomitant surgery was performed in 17 (53%) of the 32 elbows, without changing the position of the patient. The time interval between arthroscopy and the second surgery was measured since the completion in closure of arthroscopic wound and the start of surgical incision for the second surgery. The demographic data are summarized in Table 1.

**Table 1:** Demographic characteristics of patients

Age (years)	34.3 ± 9.9
Sex (*n* = 32)	
Male	24
Female	8
Side (*n* = 32)	
Right	18
Left	14
Arthroscopic time (min)	45.2 ± 98.2
Second surgery (*n* = 17)	
Ligament repair	7
Ligament reconstruction	5
Ulnar nerve transposition	4
Removal of implant	2
Carpal tunnel release	1
Shift to open (min)	6.5 ± 3.6
Follow-up (months)	17.1 ± 4.0
MEPS^a^	89.2 ± 7.2
Results (*n* = 32)^b^	
Excellent	23
Good	9

^a^ *MEPS* Mayo Elbow Performance Score

^b^The result is based on the MEPS and divided into four grades

**Fig. 1:**
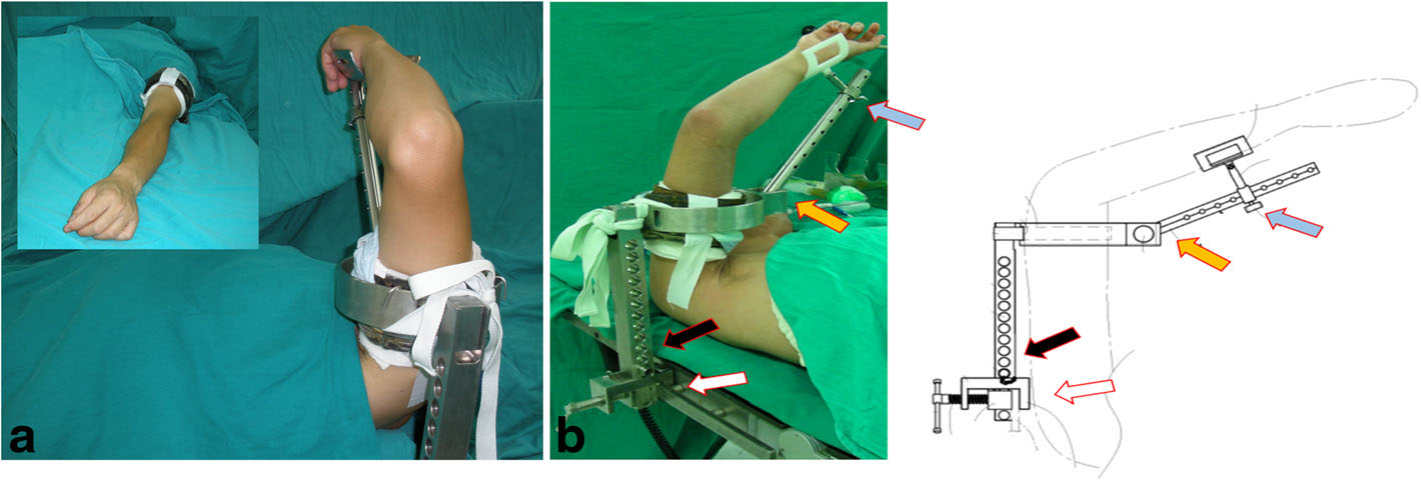
**a** The elbow was supported and secured using an adjustable arm holder. The image on the *top left* shows that the arm was lowered before conversion to open procedure, without the need for repositioning the patient. **b** The photo of the arm holder (*left*) with cartoon drawing (*right*). There are three adjustable modules pointed out with *arrows* of different colors. The *blue* and *black arrows* indicate the distal and proximal modules, which allow longitudinal adjustment according to the forearm and upper arm length, respectively. The *yellow arrow* indicates the middle module of the rotational hinge, which allows adjustment of elbow flexion. The *white arrow* indicates the fixation device, which secures the arm holder on the side bar of surgical table

**Fig. 2:**
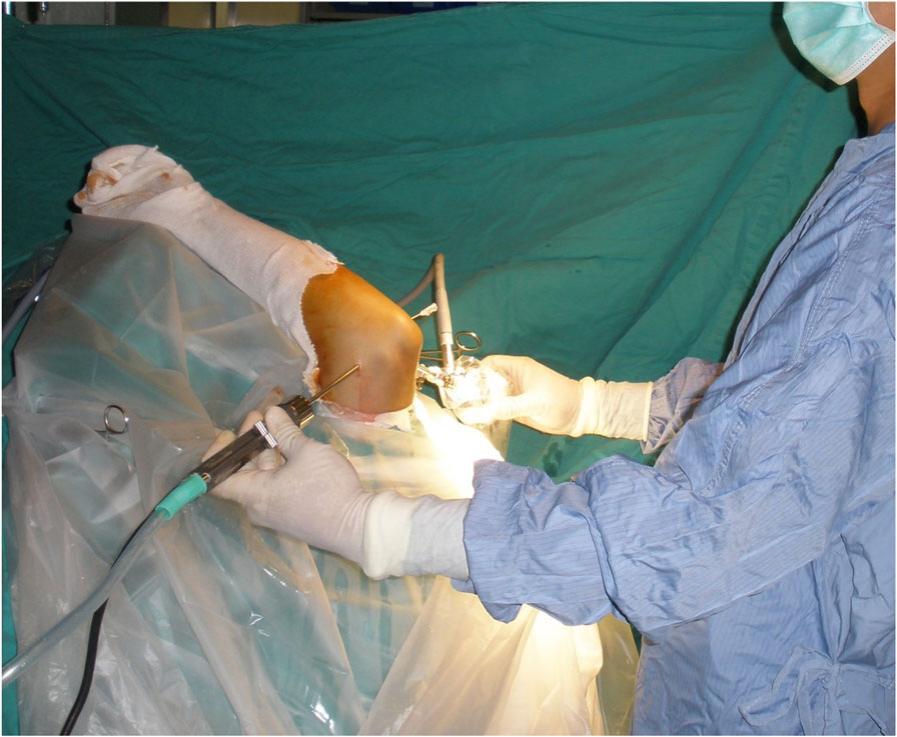
Intraoperative photograph during arthroscopy of the elbow

We performed elbow arthroscopy under either general or regional anesthesia. The patient was placed in a supine position with the elbow and the forearm supported using an arm holder (Fig. 1). A pneumatic tourniquet was applied to control intraoperative bleeding. The upper arm was secured to the arm holder using a stabilizing bandage. The arm holder, pneumatic tourniquet, and stabilizing bandage could all be easily assembled and disassembled, sterilized, and fastened to either side of the operating table, which facilitated any subsequent surgical procedure that might be required in addition to elbow arthroscopy. Because the upper arm was securely held in an upright position, the elbow could move freely and could be flexed, extended, pronated, or supinated during arthroscopy (Fig. 2). During arthroscopy, we used standard anteromedial and anterolateral portals for arthroscopic viewing and shaving. Additional working portals were created according to the location of the lesion. For treatment of posterior lesions, posterior central and lateral portals were created, while avoiding the cubital tunnel and ulnar nerve. After completion of the arthroscopic procedure, the arm holder was removed, and the arm was positioned flat on a hand table covered in sterile draping. Then, the second surgical procedure was performed, if necessary.

After surgery, all patients underwent subsequent follow-up for at least 1 year. Clinical evaluation was performed using the Mayo Elbow Performance Score (MEPS) [8].

## Results

**Fig. 3:**
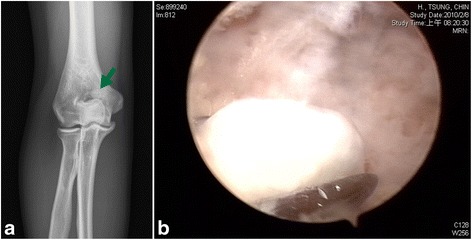
**a** Preoperative radiograph showing a loose body in the olecranon fossa (*arrow*). **b** Arthroscopic image showing removal of the loose body. **c** Arthroscopic image showing the olecranon fossa after debridement

**Fig. 4:**
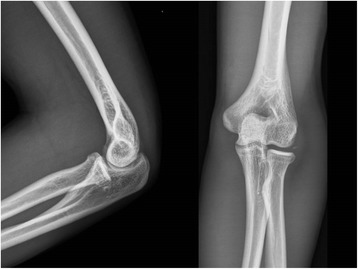
Plain radiographs failed to identify soft tissue injury and elbow instability



We performed 32 elbow arthroscopies. Primary diagnoses were made according to preoperative radiographs plus either computerized tomography (CT) scans (5 elbows) or magnetic resonance imaging (MRI, 4 elbows), including instability in the 12 elbows (7 with valgus instability and 5 with posterolateral instability), posterior impingement in the 6 elbows, loose body in the 6 elbows (2 with articular fractures), refractory lateral epicondylitis in the 4 elbows, posttraumatic arthrofibrosis in the 2 elbows, and osteochondritis dissecans (OCD) in the 2 elbows. Secondary diagnoses (Table 2) were based on additional arthroscopic findings, which were different from the primary diagnoses, including loose bodies (3), posterior impingement (2), degeneration (3), annular ligament tear (2), osteochondral defect (2), and posterolateral plica (2). Among 32 elbow arthroscopies, interventional arthroscopy (Fig. 3) was performed in 20 patients (62.5%); diagnostic arthroscopy (Fig. 4 and Additional file 1: Movie S1) before open elbow surgery was performed in 12 patients (37.5%). The average time for arthroscopy was 45.2 min (range, 34 to 78). The time interval between the end of arthroscopy and the start of the second surgery procedure averaged 6.5 min (range, 2 to 15).

**Table 2:** Diagnosis before and after arthroscopy

Diagnosis and pathology	Primary^a^	Secondary^b^
Loose body	6	3
Posterior impingement	6	2
Medial collateral ligament laxity	7	
Lateral collateral ligament laxity	5	
Lateral epicondylitis	4	
Degeneration		3
Arthrofibrosis	2	
Osteochondritis dissecans	2	
Fracture^c^	2	
Annular ligament tear		2
Osteochondral defect		2
Posterolateral plica		2

^a^Primary means clinical diagnosis before surgery

^b^Secondary means additional findings with arthroscopy

^c^Two fractures: one capitellum fracture and one lateral condyle fracture

**Additional file 1:** Video clips: Video clips during arthroscopy showing radiocapitellar rotatory instability and a tear of the annular ligament. (WMV 1.56 mb)


A second surgery was performed after arthroscopy in 17 patients (53.1%). There were a total of 19 procedures including ligament repair in 7 patients, ligament reconstruction in 5, ulnar nerve transposition in 4, and a concomitant surgical procedure other than elbow surgery in 3 patients. The second surgical procedure was performed in these 17 patients without changing their position or draping. The type of anesthesia was switched from regional to general in 2 patients during arthroscopy according to the patients’ request.

The mean duration of follow-up was 17.1 months (range, 12 to 24 months). The average clinical outcome, as assessed by MEPS, was 89.2 ± 7.2 (range, 75 to 100); all were graded as good (9 patients) or excellent (23 patients). Final motion arc averaged 113.3 ± 11.8 (range, 85 to 140) of flexion. Two patients (cases 5 and 6) had a final arc of motion less than 100°. Case 5 had a limited arc of motion from 30° to 90° of flexion before surgery; after arthroscopic release, the range of motion was from 10° to 100° of flexion. Case 6 developed osteoarthritis after previous reconstructive surgery and regained elbow motion of 10° to 95° after arthroscopic debridement. No complications were observed in the immediate postoperative period, including neurovascular injury and wound problems, or during follow-up. Two patients felt nervous and requested to switch to general anesthesia. They underwent the following surgery efficiently following laryngeal mask insertion. Three patients complained of medial elbow paresthesia along medial cutaneous nerve postoperatively. Two of them had symptoms resolved after 1 month. In 1 patient (case 3), the symptom of residual paresthesia was due to previous open surgery and did not improve or exacerbate after the index surgery.

## Discussion

Experience in performing arthroscopy and detailed knowledge of the neurovascular anatomy of the elbow are prerequisites for safe and reproducible surgical technique [9, 10]. Position of the patient in elbow arthroscopy varies; the prone and lateral decubitus positions are more frequently used, because they offer more secure arm support [11]. However, patients managed with regional block anesthesia may feel discomfort and might even be unable to tolerate these positions through the entire operation. In addition, if intraoperative conversion to general anesthesia is required, endotracheal intubation is difficult to perform and monitor when a patient is in these positions [12]. In our study, 2 of the 32 patients felt nervous and requested to switch to general anesthesia during arthroscopy. Inserting laryngeal mask with conversion of anesthesia was performed safely and efficiently with the patients in the supine position.

Instead of using a previously reported method for securing the extended arm during elbow arthroscopy in the supine position [13], we designed a method in which an arm holder supports and stabilizes the operative arm while the elbow can move freely and can be flexed, extended, pronated, and/or supinated during arthroscopy. The arm holder, which was sterilized and fastened to either side of the operation table, could be easily assembled and disassembled and allowed the surgical arm supported on a hand table without changing patient position. While many experienced surgeons can also perform subsequent elbow surgery in lateral decubitus position, supine position is more versatile in various approaches as well as intraoperative fluoroscopy for the elbow surgery [14].

Compared with Trimano and other arm-holding devices, this method has several additional advantages. During arthroscopy, the arm holder allows easy access to both the anterior and posterior compartments of the joint, and the elbow can be repositioned for arthroscopic examination and the surgical procedure according to the location of the lesion. Moreover, when the elbow is positioned in the flexed position and moved upward without any compression of the antecubital fossa, gravity increases the intraarticular working space in the anterior compartment [15], which allows greater separation between the anterior neurovascular structures and the instrumentation [16]. In the posterior compartment, working space can also be enlarged by manually extending the elbow joint during surgery. Furthermore, the range of elbow motion and stability can be evaluated intraoperatively, which we believe is essential in confirming the diagnosis and evaluating the success of surgical treatment.

Among the patients in our study, 17 patients underwent a second surgical procedure immediately after arthroscopy. In these patients, the total operative time could be reduced because this method did not require repositioning the patient or resterilization. It took less than 7 min in average in dissembling the arm holder and wrapping the hand table for open surgery. Secondary diagnosis was based on additional arthroscopic findings, which were not detected preoperatively. This is critically important when only CT scans or MRI could be available before surgery. All these pathologies not only were managed simultaneously through arthroscopy but also served as important reference to facilitate subsequent surgical procedures [17, 18]. Recent reports have documented the importance of diagnostic arthroscopy in elbow instability [19, 20] and confirmed the indications of combined arthroscopic and open surgery in chronic elbow disorders [21–23]. Common contraindications of our method in elbow arthroscopy include upper arm lesion and vascular insufficiency, which jeopardize the application of pneumatic tourniquet and wrapping bandage for keeping the arm secured on the arm holder.

Our study had some limitations that warrant consideration. Although our new method provided encouraging results, our study lacked a comparison group. In addition, the new arm holder is only a prototype and has been used for a limited number of patients. The total cost of this arm holder has not been estimated thus far. Although no complications were found with this new device, future investigation and longer follow-up are required to determine the overall benefits and clinical relevance of this method.

## Conclusions

We successfully performed elbow arthroscopy using the new arm holder with the patient in a supine position. Subsequent concomitant surgical procedures can be immediately performed without repositioning the patient. In addition, this method may facilitate diagnostic or exploratory arthroscopy before scheduled surgical procedures.

## Abbreviations

MEPS: Mayo Elbow Performance Score
OCD: Osteochondritis dissecans
